# The relationship between sleep and wake habits and academic performance in medical students: a cross-sectional study

**DOI:** 10.1186/1472-6920-12-61

**Published:** 2012-08-01

**Authors:** Ahmed S BaHammam, Abdulrahman M Alaseem, Abdulmajeed A Alzakri, Aljohara S Almeneessier, Munir M Sharif

**Affiliations:** 1 University Sleep Disorders Center, King Saud University, Box 225503, Riyadh, 11324, Saudi Arabia; 2 Department of Family and Community Medicine, College of Medicine, King Saud University, Box 225503, Riyadh, 11324, Saudi Arabia

**Keywords:** Sleep, Sleep duration, Medical students, Academic performance, School

## Abstract

**Background:**

The relationship between the sleep/wake habits and the academic performance of medical students is insufficiently addressed in the literature. This study aimed to assess the relationship between sleep habits and sleep duration with academic performance in medical students.

**Methods:**

This study was conducted between December 2009 and January 2010 at the College of Medicine, King Saud University, and included a systematic random sample of healthy medical students in the first (L1), second (L2) and third (L3) academic levels. A self-administered questionnaire was distributed to assess demographics, sleep/wake schedule, sleep habits, and sleep duration. Daytime sleepiness was evaluated using the Epworth Sleepiness Scale (ESS). School performance was stratified as “excellent” (GPA ≥3.75/5) or “average” (GPA <3.75/5).

**Results:**

The final analysis included 410 students (males: 67%). One hundred fifteen students (28%) had “excellent” performance, and 295 students (72%) had “average” performance. The “average” group had a higher ESS score and a higher percentage of students who felt sleepy during class. In contrast, the “excellent” group had an earlier bedtime and increased TST during weekdays. Subjective feeling of obtaining sufficient sleep and non-smoking were the only independent predictors of “excellent” performance.

**Conclusion:**

Decreased nocturnal sleep time, late bedtimes during weekdays and weekends and increased daytime sleepiness are negatively associated with academic performance in medical students.

## Background

Recent data have suggested that sleep is important for memory consolidation and learning [1]. Sleep deprivation results in sleepiness and impaired neurocognitive and psychomotor performance [2]. Recent reviews have indicated an important relationship between sleep patterns with learning abilities and consequent academic performance [3]. Medical students are a unique group of young adults whose academic commitments and lifestyle can impact their sleep habits and result in sleep deprivation [4]. The continuous academic demand on this group of students may result in irregular sleep/wake patterns and poor sleep quality, which may in turn negatively impact school performance [5]. Students and educators typically do not realize that sleep habits may affect academic performance [6]. In general, the relationship between sleep/wake habits and the academic performance of medical students is insufficiently addressed in the literature. Therefore, we designed this study to assess the relationship between sleep habits and sleep duration with academic performance in a large sample of healthy medical students.

## Methods

### Study population

This cross-sectional observational study was conducted between December 2009 and January 2010 at the College of Medicine, King Saud University. We targeted medical students attending classes in the first (L1), second (L2) and third (L3) academic levels. Students at these academic levels do not have night calls or shift rotations. Classes usually start at 07:30–08:00. The total number of students in the first three academic levels was 900 students (60% males). We used a systematic random sampling technique by skipping each third name on the class list (choosing numbers 1 and 2 and skipping number 3). Using the adopted systematic sampling technique, we ended with a target group of 600 students. Trained medical students and one of the authors (AB) met the medical students and explained the study objectives and protocol. During the interview, screening for chronic medical or psychiatric illnesses was performed, and informed consent was obtained. Students with known chronic medical illnesses or students who were taking drugs that cause sleepiness were excluded from the study. Participants were asked to complete a self-administered questionnaire.

Participation was voluntary, anonymous and unpaid. The study protocol was approved by the college of medicine institutional review board.

### Questionnaire

A questionnaire was designed based on previously published survey instruments to assess demographics, sleep/wake schedule, sleep habits, self-perceived sleep, sleep complaints, caffeine intake, smoking, and time allocated for daily activities and habits [4,7-9]. Caffeine intake was measured in drinks per day (8 oz. serving of coffee, espresso, tea, soft drinks, hot chocolate, or 1.5 oz. of chocolate). In addition, the students recorded their employment time outside of school. Daytime sleepiness was evaluated using the Epworth Sleepiness Scale (ESS) [10]. The ESS is a standardized validated questionnaire that assesses the likelihood that the subject will fall asleep during certain activities [10]. It consists of eight items describing different situations and activities of daily living. ESS scores range from 0–24, and based on previous studies, the upper limit of a normal score is estimated to be 10 [10]. ESS scores >10 indicate increased daytime sleepiness. Sleep/wake patterns were assessed using sleep diaries. A sleep diary is a daily log that is used to record sleep-wake pattern of individuals. Students were asked to fill out the diary each morning when they wake up, each night when they go to bed or when they nap. They were instructed to keep the diary beside their beds in order not to forget filling it. A two-week diary was completed each morning and evening by students to get estimates of bedtime, wake-up time and nap-time.

The questionnaire was pre-tested on a sample of 30 students to test the data-gathering mechanisms and to assess the legibility and reliability of the questionnaire. Thirty questionnaires were distributed, and the required modifications were performed. Moreover, to objectively verify the accuracy of the self-reported sleep duration, a random sample of 30 students was asked to wear a wrist actigraphy monitor (Mini Mitter Company, Inc., Bend, OR, USA) for three consecutive days, and the sleep duration and time were validated against the recorded sleep diaries. Correlation between actigraphy and sleep diaries were r = 0.83, p < 0.01, r = 0.79, p < 0.01 and r = 0,81, p < 0.01 for sleep onset, sleep offset and sleep duration, respectively [11].

School performance was based on the self-reported GPA (grade point average), which is a known method to define academic performance in sleep research [12-14]. The school performance was stratified as “excellent students” (GPA ≥3.75/5) or “average students” (GPA <3.75/5). This dichotomous division of academic performance has been previously used for the assessment of the relationship between sleep habits and school performance [14,15].

### Study protocol

The participants were instructed to observe their own sleep habits and other parameters described in the questionnaire during weekdays and weekends for two weeks and to complete the questionnaire on the last day of each week. In addition, sleep diaries and the ESS were completed daily for two weeks, and the mean values were used in the final analysis. Based on the data collected from sleep diaries, the following were calculated: nighttime sleep duration (total sleep time (TST)), waking time and bedtime during weekdays and weekends. The participants were contacted by trained medical students at regular intervals to verify the completeness and accuracy of data collection.

### Statistical analysis

Categorical variables were expressed as proportions, and continuous variables were expressed as the mean ± standard deviation (SD). The chi-square test was used to compare categorical data, and the *t*-test was used for continuous variables. A further analysis of the residuals was performed to identify the categories responsible for significant chi-square values [16]. To explore the association between sleep problems and habits and school performance, a univariate logistic regression model was initially used. After testing for multicollinearity among the univariate predictors, independent, significant variables were entered into a multiple logistic regression model to determine if any variable could predict school performance. The results were considered statistically significant when p ≤ 0.05. Standard statistical software (SPSS, Statistical Package for the Social Sciences, version 17.0, Chicago, IL, USA) and MS Excel were used for the data management and statistical analyses.

## Results

Of the 600 questionnaires distributed, 495 (83%) were returned. Eighty-five (17%) questionnaires were rejected for following reasons: chronic medical illnesses (n = 5), missing GPA (n = 14) and incompleteness (n = 66). Therefore, 410 questionnaires were included in the final analysis (L1, 151 (36.8%); L2, 132 (32.2%) and L3, 127 (31.0%)). There were 273 (67%) male students and 137 (33%) female students. The mean age of the participants was 20.37 ± 1.13 years. When school performance was stratified into “excellent” (GPA ≥3.75) and “average” (GPA <3.75), 115 students (28%) had “excellent” performance, and 295 students (72%) had “average” performance. There was no difference between males and females or among different academic levels in the assessed sleep parameters; therefore, we analyzed the combined data from both sexes.

Table 1 summarizes the demographics and general characteristics of the total study population as well as the “excellent” group and “average” group. The prevalence of obesity and smoking was higher in the “average” group. On the other hand, daily internet usage of more than 2 hours was significantly higher among the average group.

**Table 1:** Demographics and general characteristics of the different groups

**Variables**	***Total*** ***(n = 410)***	***“Excellent”*** ***115 (28%)***	***“Average”*** ***295 (72%)***	***P value***
Sex (Male)	273 (67)	73 (63)	200 (68)	0.405
BMI	25.01 ± 8.56	24.46 ± 11.05	25.24 ± 7.33	0.423
Obese (BMI > 30)	57 (13.9)	9 (7.8)	48 (16.3)	0.026
Smoke (Yes)	24 (5.9)	1 (0.9)	23 (7.9)	0.007
Internet Usage >2 hr	197 (48)	43 (37)	153 (52)	0.028
Job (volunteer or employee) during school days	74 (18.0)	23 (20.2)	51 (17.3)	0.506

*BMI*: Body mass index.

Table 2 presents sleep habits and characteristics of both study groups. Only 36% of the study population felt that they obtained sufficient sleep. The proportion of students who felt that they obtained sufficient sleep was significantly higher in the “excellent” group (50.0%) compared with the “average” group (30.6%).

**Table 2:** Sleep habits and characteristics of both study groups

***Variables***	***Total*** ***(n = 410)***	***“Excellent”*** ***115 (28%)***	***“Average”*** ***295 (72%)***	***P value***
Do you feel that you get sufficient sleep?	147 (36.0)	57 (50.0)	90 (30.6)	0.000
ESS	7.71 ± 3.85	6.88 ± 3.64	8.03 ± 3.89	0.007
Subjective feeling of sleepiness during classes	261 (64.9)	63 (55.3)	198 (68.8)	0.010
Weekday bedtime (24 hrs)	00.06 ± 1.58	23.76 ± 1.70	00.17 ± 1.52	0.018
Weekday wake-up time	06.15 ± 1.24	06.03 ±1.33	06.20 ± 1.20	0.196
Weekday TST (hrs)	6.04 ± 1.36	6.28 ±1.45	5.94 ± 1.31	0.026
Weekend bedtime	01.68 ± 2.20	01.14 ± 1.98	01.89 ± 2.25	0.002
Weekend wake-up time	09.69 ± 3.86	09.01 ± 3.73	09.95 ± 3.89	0.029
Weekend TST (hrs)	9.02 ± 2.45	8.96 ± 2.69	9.05 ± 2.36	0.753
Bedtime delay during weekend*	1.75 ± 1.44	1.66 ± 1.42	1.79 ± 1.46	0.447

*ESS*: Epworth sleepiness scale, *TST*: total sleep time.

*Bedtime during weekends - bedtime during weekday.

The “average” group had a higher ESS score and a higher percentage of students who felt sleepy during class (68.8% vs. 55.3%, p = 0.01). When the relationship between sleep habits and school performance was analyzed, the “excellent” group had an earlier bedtime and longer TST during weekdays. During weekends, both groups compensated for the sleep lost during weekdays by increasing the TST. Nevertheless, the bedtime and waking time remained significantly delayed in the “average” group during the weekends (Table 2). There were no statistically significant differences in the weekend sleep duration, or the study time between the “excellent” performers and the “average” performers. In addition, there was no difference in the use of caffeinated beverages, and none of the students reported using prescription or non-prescription stimulants.

Table 3 shows the independent predictors of “excellent” performance using multivariate logistic regression analysis. Subjective feeling of obtaining sufficient sleep and non-smoking remained significant predictor of “excellent” performance in a fully adjusted model.

**Table 3:** Logistic regression analysis

	***Unadjusted OR***	***Unadjusted p-value***	***Adjusted OR***	***Adjusted p-value***	***95% C.I.***
Do you feel that you get sufficient sleep?	2.135	0.000	2.184	0.002	1.343 - 3.554
Total Sleep time	1.203	0.026	1.104	0.329	0.905 - 1.348
Weekend bedtime	0.943	0.029	0.917	0.222	0.797 - 1.054
Smoking	0.102	0.007	0.122	0.044	0.016 - 0.946
BMI > 30 kg/m^2^	0.437	0.026	0.624	0.242	0.284 - 1.373

*BMI*: body mass index; *OR*: odds ration.

## Discussion

The current study demonstrates a clear association between sleep/wake habits and academic performance among medical students. Certain sleep habits were associated with lower academic performance. A late bedtime on weekdays and weekends was associated with lower academic performance. These findings agree with those of a study conducted on first-year college students showing that students with later bedtimes during weekdays and weekends had lower performance.[17] Moreover, the bedtime during weekends was delayed in both groups compared with the weekdays. Experimental studies have demonstrated that a shift in bedtime by two hours while maintaining the same sleep duration resulted in increased feelings of depression, difficulty in concentration and mood changes [5]. Previous studies have demonstrated that a shift delay in the bedtime of college students can impair the students’ academic performance [5]. Sleep duration (TST) during the weekdays was significantly longer (by around 20 minutes) in the “excellent” group. Although the difference in sleep duration may not appear remarkable, it may have physiological importance, particularly when the sleep deprivation accumulates over several days [18]. These results agree with previous reports in adolescents that demonstrated a significant impact of increased nocturnal sleep duration on academic achievement. In a sample of 3,000 adolescent students, Wolfson and Craskadon reported a significantly longer total sleep time and earlier bedtimes in students with higher grades [13]. Students reporting a “B” or better got 17–33 minutes more total sleep on average school nights and went to bed 10–50 minutes earlier than “C,” “D” and “F” students [13]. Because the students in the present study were not obtaining sufficient sleep on weekdays, they increased their sleep duration during weekends. A recent study showed that increased weekend catch-up sleep (as an indicator of insufficient weekday sleep) is associated with poor performance on objective attention tasks where the number of omission and commission errors is measured in a computerized system [19].

Although sleep deprivation affects academic performance, students who are sleep-deprived and experience academic difficulties are usually not aware of the extent to which their sleep loss can impair their ability to complete cognitive tasks. Pilcher and Walters subjected 44 college students to total sleep deprivation for one night and found that the sleep-deprived students performed significantly worse on cognitive tasks compared with students who had normal sleep [6]. Paradoxically, the sleep-deprived students who performed worse reported higher levels of estimated performance and inaccurately rated their performance as better than that of students who were not sleep-deprived [6].

Sleep deprivation may affect school performance at several levels. Daytime sleepiness and reduced levels of attention affect performance [20]. Moreover, sleep deprivation may impair memory and decision making [21]. Previous studies have linked the consolidation of higher-order implicitly learned information to the rapid eye movement (REM) stage of sleep (the dreaming stage of sleep) [22]. Previous studies reported a significant correlation between language learning efficiency and increases in the fraction of REM sleep, which suggests that learning performance may be an important factor in the relationship between information processing and REM sleep [23]. As REM sleep episodes tend to increase in the last third of the night, sleep deprivation may significantly reduce the percentage of REM sleep. In addition, poor sleep may indirectly affect performance by increasing depression, decreasing motivation and compromising health [2].

A higher proportion of the “excellent” group felt that they obtained sufficient nocturnal sleep. In addition, the subjective feeling of getting sufficient sleep was an independent predictor of “excellent” performance. Howell et al. conducted a study on college students and confirmed a correlation between poor sleep quality and academic performance [24]. The increased ESS score and the subjective feeling of sleepiness were more common among the “average” group. Sleepiness may negatively impact academic performance [25]. Daytime sleepiness has been shown to negatively affect the participation of students in extracurricular activities [26]. Daytime sleepiness is likely to be a consequence of sleep deprivation. However, circadian rhythm disorders in the form of delayed sleep phase syndrome marked by significant delays in sleep/wake cycles are common among college students and may result in increased daytime sleepiness [27]. Smoking was more common among the average group and was an independent predictor of a lower academic performance. Previous studies suggested that students who do well in school are less like to smoke. In a recent study, Morin et al in a four-year cohort study on a total of 741 adolescents reported that smoking among persistently high achievers (7.1%) was significantly lower than average achievers (15.1%) and low achievers (49.1%) [28].

The present study reports important findings; nevertheless, this type of study has inherent limitations that must be addressed. First, the self-reporting of sleep/wake habits relies on the students’ subjective accounts, which raises the possibility that the students may not have accurately reported their sleep habits. We therefore conducted a limited validation of the self-reported sleep duration data against objective actigraphy measurements. Moreover, we used sleep diaries and contacted the participants on a regular basis during the study period. Additionally, Wolfson and colleagues have demonstrated the validity of self-reported survey estimates of sleep patterns in adolescents [29]. Moreover, there are many hidden confounding variables that may influence the measurement of academic performance, such as self-concept, motivational changes, mental stress and social class. Furthermore, colinearity between the studied variables is another problem that was obvious in our preliminary analysis. Nevertheless, we tried to ameliorate that by testing for multicollinearity among the univariate predictors. At this stage, a crude analysis is required to build a solid base for future studies, especially because this area of research has not been well explored in medical students. Finally, because the study was cross-sectional, no conclusions about the long-term effects of insufficient sleep can be drawn. Although it is sensible to assume that improving the quality and pattern of sleep will contribute to the improvement of academic performance, a cause-effect relationship has not been established.

## Conclusion

This study showed that decreased nocturnal sleep time, late bedtimes during weekdays and weekends, catch-up sleep on weekends and increased daytime sleepiness are negatively associated with academic performance in medical students. The subjective feeling of obtaining sufficient sleep and the non-smoking status were the independent predictors of excellent academic performance. Educators and college authorities need to take an active role to consider sleep habits and sleep disturbances in the context of academic performance, and educate college students about good sleep hygiene. Researchers need to identify variables that lead to poor sleep quality in medical students.

## Abbreviations

BMI, Body mass index; ESS, Epworth Sleepiness Scale; GPA, Grade point average; TST, Total sleep time.

## Competing interests

The author(s) declare that they have no competing interests.

## Authors’ contributions

AB: conception, design, data analysis and manuscript writing. AMA and AAA: conception, design and data collection. ASA: data analysis and manuscript writing. MS: design, data analysis and manuscript writing. All authors read and approved the final manuscript.

## Authors’ information

AB: sleep medicine specialist

AMA and AAA: medical students

ASA: medical education specialist

MS: data manager and statistician

## Source of funding

This project was partially funded by The National Plan for Sciences and Technology (King Saud University and King Abdulaziz City for Science and Technology).

## Pre-publication history

The pre-publication history for this paper can be accessed here:

http://www.biomedcentral.com/1472-6920/12/61/prepub
